# Parental acceptance of advanced behaviour management techniques in paediatric dentistry in families with different cultural background

**DOI:** 10.1007/s40368-021-00607-4

**Published:** 2021-03-25

**Authors:** L. Al Zoubi, J. Schmoeckel, M. Mustafa Ali, CH. Splieth

**Affiliations:** 1 Department of Preventive and Paediatric Dentistry, University of Greifswald, Walther-Rathenau-Straße 42, 17475 Greifswald, Germany; 2 Department of Pediatric Dentistry and Orthodontics, Taibah University, Medina, Saudi Arabia

**Keywords:** Nitrous oxide sedation, Parental acceptance, Advanced behaviour management techniques, General anaesthesia

## Abstract

**Purpose:**

To investigate the differences of parental acceptance of advanced behaviour management techniques (BMT) in different cultural backgrounds (Germany vs. Jordan).

**Methods:**

A convenience sample of 100 parents each of paediatric patients at the University of Greifswald/Germany and Jordan University/Jordan completed a questionnaire to rate their acceptance of four advanced BMT (passive restraint, active restraint, nitrous oxide sedation, and general anaesthesia) using a five-point Likert scale.

**Results:**

In both groups, nitrous oxide sedation was the most accepted advanced BMT (mean 3.78 ± 1.34/3.22 ± 1.50, respectively). The least acceptable technique in Germany was passive restraint (2.05 ± 1.18) and in Jordan general anaesthesia (2.11 ± 1.30). The parents in Germany are significantly more accepting of nitrous oxide sedation than are parents in Jordan (*p* = 0.010), while parents in Jordan are more willing to accept passive restraint (*p* = 0.001). The acceptance of all advanced BMT increased significantly in both groups when the treatment was urgent.

**Conclusions:**

Parental cultural background and the urgency of the treatment affect the acceptance of different BMT. Moreover, the parental attitude to the pharmacological technique has changed, as nitrous oxide sedation generally appears to be the most preferred advanced technique in both groups.

## Introduction

Managing anxious, uncooperative children can be one of the most challenging aspects of paediatric dentistry (Gazal et al. 2016). Especially, a high percentage of children who are treated in specialised dental clinics have dental fear and anxiety (DFA) (Anthonappa et al. 2017). Dental fear is an emotional response to a specific external stimulus, such as needles during dental treatment, while dental anxiety is a nonspecific feeling of apprehension (Haliti and Juric 2017).

This is the reason for the importance of behaviour management techniques (BMT), which are considered integral components in paediatric dentistry, to alleviate fear and anxiety, efficiently deliver effective dental treatment to the child to achieve a better outcome, and promote a positive dental attitude towards oral health care (Elango et al. 2012; Alammouri 2006). The behaviour management techniques can be divided into (1) basic behaviour techniques, e.g., tell-show-do, distraction, positive reinforcement, voice control, and parental presence/absence, and (2) advanced behaviour techniques which includes protective stabilisation (active and passive restraint), sedation, and general anaesthesia (GA) (AAPD 2016).

The choice of BMT is not made by the dentist alone; the parent and the child should participate in the decision making process (Havelka et al. 1992). Additionally, the parents are legally and ethically responsible for their children, so the selection of BMT is made with active involvement and informed consent of the parents (Boka et al. 2014). Therefore, the knowledge about the parental attitude towards different BMT is considered an important approach in paediatric dentistry to build up parents’ trust to promote optimal treatment of the child (Lawrence et al. 1991; Eaton et al. 2005; Patel et al. 2016).

There are many factors that potentially play a role in parental acceptance of a particular BMT, such as the treatment need and its urgency, cooperation level of the child, and socio-economic status of the parents (Peretz et al. 2013; Patel et al. 2016). Several studies showed different parental attitudes towards BMT, and it is interesting to note that the acceptability of the techniques has changed over time. In 1984, in USA, voice control, positive reinforcement, and tell-show-do were considered as acceptable techniques for all dental procedures, while pharmacological techniques, which include sedation and GA, were acceptable only for emergency extraction and restoration (Fields et al. 1984). In another study in Thailand 2002, the parents rated tell-show-do, positive reinforcement, distraction, and papoose board as the most acceptable techniques. In contrast, sedation and GA were considered the least acceptable techniques (Kamolmatayakul and Nukaw 2002). While the acceptance of pharmacological techniques has increased in USA (2005), Sedation along with the tell-show-do technique were rated as the most acceptable techniques. On the other hand, passive restraint (papoose board) and hand-over-mouth technique were then considered the least acceptable techniques (Eaton et al. 2005). Furthermore, in Spain, in 2010, the most acceptable techniques were tell-show-do, followed by active restraint, nitrous oxide (N_2_O) sedation, and GA, while the least accepted were the hand-over-mouth technique and papoose board (Leon et al. 2010). In another study in India (2016), showed that sedation and GA were rated as acceptable techniques (Acharya 2017). This means that parental attitude towards different behaviour management techniques in paediatric dentistry is not fixed and changes over time and is affected by social and cultural changes (Jafarzadeh et al. 2015). This is why, it is important to regularly reassessment of the acceptance of the parents regarding different BMT to determine the most appropriate technique during treatment (Boka et al. 2014). Although some studies have assessed parental acceptance of various behaviour management techniques in paediatric dentistry, only a limited number of studies have evaluated parental acceptance of advanced BMT. Moreover, to our knowledge, there is no study comparing the parental acceptance of advanced BMT between two countries with clearly different cultures, such as Germany and Jordan.

Therefore, the purpose of this study was to investigate the potential differences of parental acceptance of advanced behaviour management techniques between parents seeking dental treatment for their child at the University of Greifswald in Germany vs Jordan University in Jordan.

## Materials and methods

This study was a cross-sectional questionnaire survey, distributed among two convenience samples of 100 parents each accompanying their children for treatment at the Department of Paediatric Dentistry at the University of Greifswald/Germany or Jordan University Hospital/Jordan.

### Ethical approval

Permission to carry out the study was obtained from the ethics committee of the University of Greifswald (number BB 081/16) and from the Institutional Review Board (IRB) in Jordan (number 21/2016).

The inclusion criteria for participation were: parenthood, literacy, and willingness to participate. Parents of children with special health care needs were excluded from the study.

### Data collection

The parents filled out a questionnaire consisting of two parts. The first part requested information about parent’s personal data such as gender and educational level. In the second part of the survey, the parents were asked to determine their acceptance of each advanced BMT in normal treatment and in an emergency situation. Advanced BMT included passive restraint (partial or complete stabilisation of the child during dental treatment by a restrictive device such as papoose board), active restraint (partial or complete stabilisation of the child by the practitioner, staff, or the parent during dental treatment), N_2_O sedation (the administration of N_2_O gas via a mask during dental treatment to decrease anxiety, while the child stays conscious and can hear and respond to any request), and GA (controlled state of unconsciousness during dental treatment). The emergency situation was defined as a child with a toothache or dental trauma.

The techniques were explained in the questionnaire by providing the definition of each technique according to the current clinical guidelines on behaviour management techniques by the American Academy of Pediatric Dentistry (AAPD 2016) and by two additional photos for each technique except GA. The photos in the questionnaire were taken at the Department of Preventive and Paediatric Dentistry in the University of Greifswald. Consent for the use of the photos for research and educational purposes was obtained from the parents of each child shown in the photos.

The acceptance rating was determined on a five-point Likert-type scale ranging from 1 (highly unacceptable) to 5 (highly acceptable). The study was conducted according to the Declaration of Helsinki (World Medical 2013). The parents in this study received verbal and written information about the nature and purpose of the study, and all participants were made aware that participation was voluntary.

### Statistical analysis

Data entry and analysis were performed using the SPSS statistical package (version 20). Frequency and percentage tables were generated to present the descriptive statistics, and mean ± standard deviation (SD) was used to describe the acceptance of each advanced BMT.

A paired sample *t *test was conducted to find any significant differences between the parents’ gender, emergency situations and the acceptance levels of each advanced BMT. ANOVA was applied to determine if significant differences existed between the acceptance of each advanced BMT and the educational level of the parents. If statistically significant differences were found, a post hoc test was used to identify significant differences in different educational-level groups. Post hoc power analysis was conducted using (G*power program version 3.1) to measure the power of the study. The level of significance was defined as *p* ≤ 0.05.

## Results

200 parents agreed to participate in the study. 100 parents accompanying their children to the Department of Paediatric Dentistry at the University of Greifswald in Germany, and 100 parents accompanying their children to the Department of Paediatric Dentistry at the Jordan University Hospital in Jordan answered the questionnaire. The available demographic data of the parents for each group are summarized in Table 1.

**Table 1:** Demographic characteristics of the parents

Questionnaire	University of Greifswald Germany *N* = 100	Jordan University Jordan *N* = 100
	Frequency (percentage)	Frequency (percentage)
Parent	*N* = 100	*N* = 99
Mother	74 (74%)	84 (84.8%)
Father	26 (26%)	15 (15.2%)
Parental educational level	*N* = 96	*N* = 100
Primary school	1 (1%)	4 (4%)
Middle school	42 (43.8%)	8 (8%)
High school	19 (19.8%)	23 (23%)
Vocational training (Diploma)	19 (19.8%)	22 (22%)
Bachelor’s degree	15 (15.6%)	43 (43.0%)

### Parental acceptance of advanced BMT

The means and standard deviations for the four advanced behaviour management techniques for both groups are shown in Table 2. The paired t tests indicated that passive restraint was significantly more accepted in the Jordan University group than in the University of Greifswald group (*p* = 0.001). In contrast, the parents in the University of Greifswald group were significantly more accepting of N_2_O sedation than were the parents in the Jordan University group (*p* = 0.010).

**Table 2:** Parental acceptance of advanced behaviour management techniques

Behaviour management techniques	University of Greifswald Germany	Jordan University Jordan	*p* value
	Mean (± SD)	Mean (± SD)	
Passive restraint	2.05 (± 1.18)	2.52 (± 1.50)	0.001*
Active restraint	2.99 (± 1.32)	3.08 (± 1.33)	0.855
N_2_O sedation	3.78 (± 1.34)	3.22 (± 1.50)	0.010*
General anaesthesia	2.78 (± 1.38)	2.11 (± 1.30)	0.430

*Significant (*p* ≤ 0.05)

For the University of Greifswald group, N_2_O sedation (mean 3.78 ± 1.34) was rated as the most accepted technique, followed in order of decreasing acceptance by active restraint, GA, and passive restraint, which was the least accepted technique (mean 2.05 ± 1.18). However, in the Jordan University group, the most accepted technique was N_2_O sedation (mean 3.22 ± 1.50) followed by active restraint, passive restraint, and general anaesthesia (mean 2.11 ± 1.30).

It is noteworthy that if the child were in pain and the treatment considered urgent, parents in both countries were significantly more accepting of all advanced behaviour management techniques, except for active restraint in the group of parents in the University of Greifswald group (*p* < 0.001, *t* tests, Table 3).

**Table 3:** Effect of demographic characteristics on parental acceptance in the University of Greifswald and Jordan University groups estimated by t test/ANOVA

	Passive restraint		Active restraint		N_2_O sedation		General anaesthesia	
	Mean	*p* value	Mean	*p *value	Mean	*p *value	Mean	*p *value
University of Greifswald								
Parent								
Mother	2.03	0.567	3.16	0.249	3.88	0.550	2.84	0.761
Father	2.12		2.50		3.52		2.62	
Educational level								
Primary school	3.00	0.579	4.00	0.435	4.00	0.116	2.00	0.087
Middle school	2.05		2.76		3.95		3.17	
High school	1.68		2.89		4.16		2.26	
Vocational training	2.16		3		3.11		2.72	
Bachelor’s degree	2.20		3.47		3.43		2.27	
When the treatment is urgent	2.6	0.000*	3.17	0.075	4.07	0.000*	3.42	0.000*
Jordan University								
Parent								
Mother	2.40	0.096	3.02	0.352	3.30	0.430	2.07	0.854
Father	3.13		3.40		2.64		2.27	
Educational level								
Primary school	3.50	0.605	4.00	0.042*	2.25	0.732	2.75	0.699
Middle school	2.75		2.00		3.13		2.50	
High school	2.65		2.78		3.26		2.17	
Vocational training	2.50		3.36		3.41		1.95	
Bachelor’s degree	2.33		3.22		3.22		2.02	
When treatment is urgent	3.23	0.000*	3.43	0.000*	3.51	0.000*	3.29	0.000*

*Significant (*p* ≤ 0.05)

### Effect of demographic characteristics on the acceptance of advanced BMT

Parents’ gender did not significantly affect the acceptability of all advanced BMT in Germany or in Jordan (*t* test).

Additionally, for the University of Greifswald group, there was no significant relation between the educational level of the parents and the acceptance of all advanced BMT (Table 3). However, in the Jordan University group, ANOVA indicated a significant relationship between educational level of the parents and their acceptance of active restraint. The significant differences in acceptance were found between the parents with a primary school education vs. those with a middle school education, with higher acceptance of active restrain in the primary school group (*p* < 0.05, post hoc test, Table 3). However, this finding should be regarded with caution due to the low number of parents (*N* = 4) finishing primary school and middle school (*N* = 8) in the Jordan University group.

Moreover, there was no significant relationship between the educational level of the parents and the acceptance of passive restraint, N_2_O sedation, and GA in the Jordan University group.

## Discussion

The aim of the present study was to assess the difference in acceptance of advanced BMT for dental treatment between parents at the University of Greifswald in Germany and at Jordan University in Jordan. Post hoc power analysis was measured in this study with a median power of 0.74. However, a power of 0.8 is recommended to obtain statistical power (Cohen 1988) Nevertheless, the results point in a clear direction and only the statistical power of these observations is slightly reduced. The study sample may not be representative of the population in Germany or in Jordan, as it reflects the typical parents attending a specialised paediatric dental clinic with their children for their child's dental needs. Especially, the fact that first visits for children’s emergency treatment are significantly more common in university-based paediatric clinics than in community-based clinics (*p* < 0.001) shows that these children and parents are a relevant target group (Meyer et al. 2017).

Moreover, one has to consider that the techniques were explained verbally to the parents with the additional use of photos and a written explanation, as they usually had no actual experience with any technique. These were similar to what was used in the studies by Peretz et al. (2013) and by Brand et al. (1995). Moreover, Allen et al. (1995) and Mehrysa et al. (2014) showed no significant difference in the parental acceptance of BMT when parents were informed about BMT by written information, oral interview, or video explanation. However, it could still be a confounding variable that may affect the parental acceptance to different advanced BMT. Furthermore, broader and comprehensive studies are required to evaluate the effect of previous experience of an advanced BMT on parental acceptance.

Though some authors have studied the parental acceptance of different BMT in paediatric dentistry. Acceptance of various behaviour management techniques has changed over the years; for example, parents today more often accept pharmacological management, such as N_2_O sedation and GA (Lawrence et al. 1991; Havelka et al. 1992; Scott and Garcia−Godoy 1998; Eaton et al. 2005; Patel et al. 2016).

Apparently, the parents in this study rated N_2_O sedation as the most acceptable advanced BMT not only at the University of Greifswald, but also at Jordan University. N_2_O sedation is considered a safe, convenient, and effective method to control anxiety during dental treatment (Kanagasundaram et al. 2001). This finding agrees with the other studies conducted in 2005 and 2016, showing that most parents in the USA ranked sedation as the most acceptable BMT (Eaton et al. 2005; Patel et al. 2016). However, several studies conducted in the Middle East (Kuwait, Saudi Arabia) considered nitrous oxide sedation as an unacceptable technique (Abushal and Adenubi 2003; Muhammad et al. 2011). Therefore, the individual dentist and her/his way of explaining to the parents, e.g., N_2_O sedation in detail, may play a role in enhancing the parental acceptance in terms of advantages and adverse effects. Whenever it was suitable and indicated, this was confirmed by the other studies (Lawrence et al. 1991; Scott and Garcia−Godoy 1998; Abushal and Adenubi 2003).

While the pharmacological approach with nitrous oxide sedation was rated as the most acceptable advanced BMT in the Jordan University group, GA was rated as the least acceptable technique. At the same time, active restraint and passive restraint were considered more acceptable than GA. This stands in partial contrast to the findings in a study conducted in Jordan in 2006, which revealed that the majority of parents refused nitrous oxide sedation and general anaesthesia. The author of that study suggests that parents’ low acceptance of these techniques may be due to an unclear understanding of their respective benefits and risks, and that they are most likely unfamiliar with these techniques due to their high costs (Alammouri 2006).

Similar to other studies (Patel et al. 2016; Boka et al. 2014; Elango et al. 2012; Lawrence et al. 1991; Al Zoubi et al. 2019), the current study found that passive restraint was ranked by parents in the University of Greifswald group as the least acceptable advanced BMT. The Academy of Dental Learning and OSHA Training in USA considered passive restraint as an aggressive technique that might have serious consequences such as physical injury to the child, parent or the dentist, and possibly overwhelming psychological stress which may lead to dental phobia (Mary Oeding 2015).

Results from this study indicated that passive restraint was significantly less accepted in the University of Greifswald group than in the Jordan University group (*p* = 0.001).

Furthermore, the parents in the University of Greifswald group accepted N_2_O sedation to a significantly greater extent in comparison to the parents in the Jordan University group (*p* = 0.001). The different outcomes between the two samples from Germany and Jordan are probably due to cultural and socio-economic differences.

Results have also shown higher parental acceptance of advanced BMT in both groups when the treatment is urgent (e.g., pain or dental trauma). This is consistent with the other studies, reporting that parents are more willing to accept advanced BMT in emergency situations or when the child is experiencing pain or discomfort (Patel et al. 2016; Fields et al. 1984; Al Zoubi et al. 2019).

This study found that there is no correlation between the parental acceptance of different advanced BMT and the educational level of the parents attending the clinics at the University of Greifswald. In the present study, the majority of the parents in the University of Greifswald group reported earning a middle school diploma (43.8%), while only 15.6% had a bachelor’s degree. Knowing that there is a relationship between caries risk of the child and parental educational level in Germany (DAJ 2017; Schmoeckel et al. 2015), this may affect the outcome. Children whose parents have a high educational level demonstrate a lower caries risk, and consequently, fewer dental appointments in comparison to children of less-educated parents (Cianetti et al. 2017; Rajab et al. 2014). However, the relationship between parental educational level and their acceptance of different BMT is still unclear. Some studies found differences in attitudes between parents of different educational levels (Fields et al. 1984; Havelka et al. 1992), while others reported no correlation between the educational level of the parents and their acceptance of different BMT (Eaton et al. 2005; Muhammad et al. 2011).

This study employed convenience sampling of parents accompanying their child to the dental clinics at the University of Greifswald/Germany and at Jordan University/Jordan. The sample of this study may thus not be representative of parents in these countries, but is likely vicarious of parents seeking specialised dental treatment for their child. Further studies with a slightly larger sample size to obtain a statistical power of 0.80 level are recommended (Cohen 1988).

## Conclusion

Considering the limitations of the study, the following conclusions can be drawn:

1. Cultural background and the urgency of the treatment influence the acceptance of advanced behaviour management techniques in paediatric dentistry.
2. The parental attitude to the pharmacological technique has changed compared to other studies, especially for nitrous oxide sedation, as nitrous oxide generally appears to be the most preferred advanced technique in Germany and Jordan.
